# The central role of EMT in tumor progression: mechanistic drivers, biomarker discovery, and therapeutic horizons

**DOI:** 10.3389/fphar.2026.1820481

**Published:** 2026-05-08

**Authors:** Prasanna Srinivasan Ramalingam, Baby Ilma, Md Sadique Hussain, Vikash Jakhmola, Rabab Fatima, Amita Joshi Rana, Mohit Agrawal, Sumel Ashique, Janaki Ramaiah Mekala, Sivakumar Arumugam

**Affiliations:** 1 Protein Engineering Lab, School of Biosciences and Technology, Vellore Institute of Technology, Vellore, India; 2 Sharda School of Pharmacy, Sharda University, Greater Noida, India; 3 Uttaranchal Institute of Pharmaceutical Sciences, Uttaranchal University, Dehradun, India; 4 School of Health Sciences and Technology, UPES, Energy Acres, Dehradun, India; 5 College of Pharmacy, Graphic Era Hill University, Bhimtal, India; 6 Centre for Promotion of Research, Graphic Era (Deemed to be) University, Dehradun, India; 7 Department of Pharmacology, School of Medical and Allied Sciences, K.R. Mangalam University, Gurugram, India; 8 Department of Pharmaceutical Technology, Bharat Technology, Uluberia, India; 9 Department of Integrative Biology, School of Biosciences and Technology, Vellore Institute of Technology, Vellore, India

**Keywords:** artificial intelligence, biomarker, diagnosis, epithelial-mesenchymal transition, multi-omics, therapeutics

## Abstract

The epithelial-mesenchymal transition (EMT) is a central plasticity program in cancer. It enables carcinoma cells to acquire migratory, invasive, immunomodulatory, and therapy-resistant phenotypes. As a result, EMT is a key driver of metastatic progression and poor prognosis. This overview summarizes recent mechanistic innovations across EMT-inducing transcription factors (SNAIL, SLUG, TWIST, ZEB) and their interactions with major signaling cascades, including TGF-β/SMAD, Wnt/β-catenin, Notch, PI3K/AKT/mTOR, and Hippo (YAP/TAZ). Together, these cascades integrate cues from the TME to sustain partial/hybrid EMT states and maintain cancer stemness. We present a review of epigenetic and post-transcriptional regulation of EMT (DNA methylation, histone regulation, and non-coding RNAs) and their role in reversible states transitions and drug tolerance. The translational section highlights advance in biomarker development and limitations of the static, single-timepoint assays in capturing EMT dynamics. We describe the possibilities of longitudinal, multimodal assessment, such as liquid biopsy, spatial profiling, and integrative multi-omics, for real-time monitoring of EMT states. Moreover, we explore the therapeutic opportunities involving epigenetic regulators, RNA-based interventions, EMT-immune crosstalk target, and selected natural products that modulate EMT circuits. Lastly, we propose a precision-oncology model that can consolidate the use of EMT-state stratification (epithelial-predominant, hybrid-mesenchymal, mesenchymal-predominant), adaptive monitoring, and rational combination therapies (with immunotherapy, ferroptosis inducers, and targeted agents) to overcome metastasis and resistance. Taken together, positioning EMT as a continuous, druggable spectrum, over binary switch, allows patient stratification using biomarkers and supports the development of next-generation interventions to reduce the risk of metastasis and enhance long-term clinical outcomes.

## 1 Introduction

Cancer is an inherently heterogeneous and dynamic disorder, exhibiting variability not only across tumor types and subtypes but also within individual tumors. This heterogeneity manifests both spatially and temporally, driven by clonal evolution and sequential mutations (Zhang et al., 2022). Metastasis is the leading cause of cancer-related deaths, which is reported to cause about 90% of such deaths. One hallmark of cancer is the activation of invasion and metastasis. Actually, one of the principal features of cancer malignancy remains the spread from the primary site and colonization of distant organs to form metastases (Dagogo-Jack and Shaw, 2018). Although the improvements in the early detection and initial treatment of tumors have notably increased the survival outcomes (Hussain et al., 2025), the therapeutic efficacy against metastatic tumors remains limited. The metastatic cascade largely entails a cascade of coordinated events that include detachment of tumor cells from the primary lesion, acquisition of migratory properties, intravasation into the circulatory or lymphatic systems, survival in transit, extravasation, and eventual colonization at distant sites (Fares et al., 2020). These processes are regulated by complex molecular signaling networks and dynamic crosstalk between tumor cells and their microenvironment. Important elements of this microenvironment like extracellular matrix organization, growth factors, cytokines, chemokines, and matrix metalloproteinases, in combination, regulate cellular plasticity, motility, and metastatic potential (Steeg, 2016). Besides genetic diversity, cancer cell plasticity also enables reversible adjustments to microenvironmental signals via the epithelial-mesenchymal transition (EMT) and its inverse, mesenchymal-epithelial transition (MET) (Brabletz et al., 2018).

These processes are heavily regulated by epigenetic mechanisms rather than being mutation-driven, which makes the process very dynamic and reversible. Mesenchymal cancer cells tend to have an increased resistance to apoptosis, which enables them to survive apoptosis following detachment of the extracellular matrix, a phenomenon referred to as anoikis resistance (Kim et al., 2012). They also demonstrate improved resistance to standard chemotherapy and targeted therapies, as EMT programs frequently activate survival pathways and alter drug uptake mechanisms. Furthermore, it has been proposed that EMT may play a role in maintenance of the cancer stem cell (CSC) phenotype that offers cancer cells the property to regenerate themselves as well as proliferate into tumors in secondary locations.

This review aims to provide a consistent and integrated overview of EMT as a major driver of cancer progression, metastasis, and resistance to treatment. This article addresses the role of EMT on tumor heterogeneity, cancer stemness and immune evasion by integrating mechanistic insights from molecular, cellular, and microenvironmental studies. The review further seeks to highlight emerging biomarkers that capture the dynamic and reversible nature of EMT, enabling improved stratification of patients and guiding precision therapeutic interventions. Furthermore, the review succinctly discusses the major pathways namely TGF-β/SMAD, Wnt/β-catenin, and Notch respectively that cooperate with TME cues such as hypoxia, cytokines, and extracellular matrix remodeling to sustain EMT and its intermediate hybrid states.

## 2 EMT in cancer biology

Hypoxia within the TME is a major driver of tumor growth and progression. EMT not only plays a critical role in wound healing and embryogenesis but also invokes the cascades related to tumor invasion and metastasis. It has been proven that hypoxia is the chief controller of pathological EMT and is therefore a governing factor and a mechanistic link between hypoxic conditions and cancer development (Chen et al., 2023). The entire cascade of EMT is a hallmark for highly conserved cellular programs essentially required for embryogenesis, tissue remodelling as well as wound healing that perhaps become aberrantly reactivated during cancer stages. This process describes a reversible transformation of polarized epithelial cells into motile mesenchymal cells through coordinated changes in gene expression, cellular architecture, and function. EMT is essential to gastrulation, neural crest differentiation, and organogenesis in which loss of apical-basal polarity and downregulation of adhesion molecules such as E-cadherin in addition to gain massive migration capacity to fill diversified sites there, respectively. In normal physiology, EMT is closely associated with wound healing in which the mobility of marginal epithelial cells in the wound area is driven in a more detailed way and close the tissue gaps beneath the skin. This is followed up by mesenchymal-epithelial transition (MET) to restore the targeted tissue structure. These are tightly regulated spatial and temporal processes that ensure proper tissue development and repair (Das et al., 2019).

The entire process is largely controlled by master transcription factors, such as snail1/2, twist1/2, and zeb1/2. These factors silence epithelial genes and activate mesenchymal programs. A key molecular signature is the downregulation of E-cadherin. This disrupts adherens junctions and dissolves epithelial architecture. As a result, β-catenin is released and becomes transcriptionally active (Huang et al., 2022). At the same time, mesenchymal markers of cellular reorganization include N-cadherin, vimentin, fibronectin, and α-smooth muscle actin. Cadherin switching reshapes cellular adhesion properties. This promotes heterotypic stromal and inhibiting homotypic epithelial interactions. Cytoskeletal reorganization includes vimentin and augmented actin dynamics. Moreover, the basement membrane degradation produced by multiple MMPs through the convergent manner of numerous signaling pathways supporting EMT including TGF-β, Notch and multiple receptor tyrosine kinases combine myriad microenvironmental signals with diverse intrinsic cellular responses (Imodoye et al., 2021).

EMT exists as a dynamic spectrum rather than a binary switch. Full EMT represents a complete transition in which cells lose epithelial characteristics and acquire a fully mesenchymal phenotype; however, this state is not commonly observed in clinical conditions. Instead, cancer cells more often undergo partial EMT (p-EMT), in which they retain epithelial features while simultaneously expressing mesenchymal traits, resulting in the co-expression of both epithelial and mesenchymal markers (Li D. et al., 2023). This kind of epithelial/mesenchymal hybrid cells are likely to be placed in an intermediate position in the EMT spectrum with collective migration phenotype and increased metastatic capacity. This may also be supported by single-cell studies that reveal a fine intratumoral heterogeneity with individual cells in a discrete EMT spectrum that reflects this plasticity (Sample et al., 2023). Moreover, the ability to transition between states is primarily governed by numerous epigenetic processes such as histone modifications as well as DNA methylation pattern rather than an irreversible genetic change. In cancer, EMT gives an invasive and metastatic capability, with tumor cells with invasive potential often displaying p-EMT because it results in facilitating intravasation into vasculature, anoikis resistance in circulation and extravasation at distant sites.

There is an important correlation between EMT and CSC property, linking cellular plasticity to treatment failure. EMT induction confers stem-like characteristics including self-renewal capacity, tumor-initiating ability, and stemness marker expression (Singh and Settleman, 2010). This EMT stemness axis is regulated by shared transcriptional networks involving pluripotency factors such as SOX2, OCT4, and NANOG. Cancer cells in mesenchymal or hybrid epithelial/mesenchymal states show enhanced resistance to chemotherapy, targeted therapies, and radiation. This resistance arises through multiple mechanisms. These include ABC transporter upregulation, activation of PI3K/Akt and MAPK survival pathway, enhanced DNA repair, metabolic reprogramming toward oxidative phosphorylation. Additionally, these cells evade immune surveillance via MHC class I downregulation and PD-L1 upregulation (Müller et al., 2016). This dynamic reversibility complicates therapeutic targeting but represents a vulnerability for intervention strategies aimed at disrupting metastatic cascades and overcoming treatment resistance.

## 3 Mechanistic drivers of EMT in tumor progression

### 3.1 Transcription factors orchestrating EMT

**FIGURE 1:**
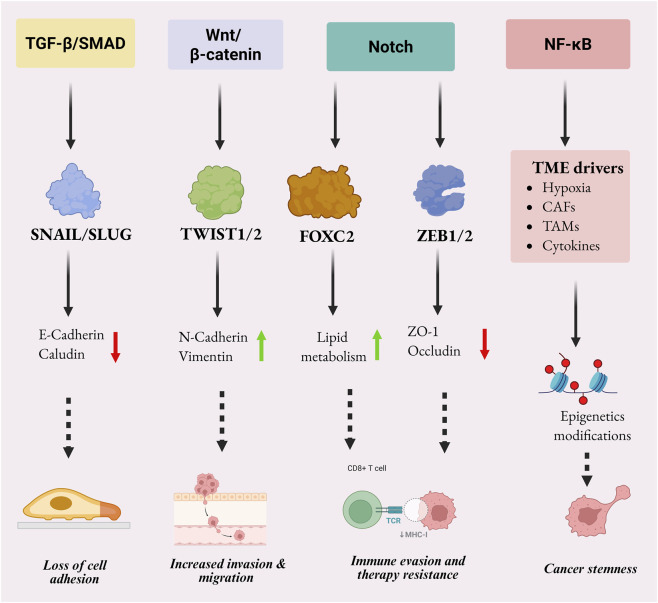
Schematic overview of EMT-regulatory signaling in cancer, showing how TGF-β/SMAD, Wnt/β-catenin, Notch, and NF-κB pathways converge on EMT transcription factors, including SNAIL/SLUG, TWIST1/2, FOXC2, and ZEB1/2. These programs drive loss of epithelial traits, acquisition of mesenchymal features, and downstream effects such as invasion, immune evasion, therapy resistance, and cancer stemness, with additional modulation by TME drivers and epigenetic changes. Overall, it highlights the hierarchical flow from upstream signals to EMT effectors and integrates pathway crosstalk, junctional remodeling, and phenotypic plasticity. Image created with BioRender.com.

Transcription factors, including Snail, Slug, Twist, and ZEB1/2, repress the epithelial markers (E-cadherin) and induce cellular programs linked to invasion, migration, and metastasis, coordinating EMT as shown in Figure 1. Of them, Snail is a key regulator in various cancers. The expression of GPX2 in NSCLC was high in tumor tissues and associated with lymph node metastasis, greater tumor size, and high TNM stage. Functional analysis indicated that the overexpression of GPX2 intensified EMT, migration and invasion but the knockdown inhibited such effects *in vitro* and *in vivo*. Mechanistically, GPX2 inhibited accumulating reactive oxygen species (ROS) and activating PI3K/AKT/mTOR, which increased Snail (Peng et al., 2023). Following the significance of Snail in EMT, a report on gastric cancer showed that SMC1A was significantly over-expressed in tissues and cell lines, and high expression was significantly associated with poor overall survival. The effect of SMC1A overexpression on proliferation, migration, and invasion were found functional and were reversed by knockdown in functional assays. SMC1A upregulated Snail expression in a mechanistic manner and, therefore, resulted in EMT and tumor progression (Liu et al., 2023). The importance of Snail in controlling the tumor microenvironment was reported in colorectal cancer where its overexpression was associated with lung metastasis and poor prognosis. Snail augmented EMT and M2-type tumor-associated macrophages (TAMs) secretion triggered by mesenchymal cells and boosted tumor invasion and metastasis. The *in vivo* experiments in nude and NOD-SCID mice indicated that the Snail-mediated CXCL2-mediated TAM infiltration facilitated pulmonary metastasis (Bao et al., 2022).

Other EMT transcription factors, including Slug and Twist, often function cooperatively with Snail to reinforce mesenchymal programs and confer therapy resistance. In head and neck squamous cell carcinoma (HNSCC), high Slug expression correlated with p-EMT, enhanced cell motility, reduced oxidative phosphorylation, and poor overall survival (Schinke et al., 2022). In triple-negative breast cancer, cordycepin treatment downregulated TWIST1, SLUG, SNAIL1, and ZEB1, restored E-cadherin expression, and suppressed tumor growth, migration, invasion, and metastasis in both *in vitro* and *in vivo* models (Wei et al., 2022). In prostate cancer, elevated Twist, Slug, and Snail expression correlated with hypoxia and angiogenesis, predicting earlier recurrence and shorter cancer-specific survival, with co-expression showing additive adverse effects (HR = 3.0 for Slug in E-cadherin low tumors, p = 0.041) (Børretzen et al., 2021). In oral squamous cell carcinoma, histamine receptor H1 upregulated the Snail/Slug axis via ADAM9, promoting EMT and lymph node metastasis, whereas dual HRH1/STAT3 blockade suppressed these effects (Ding Y.-F. et al., 2025).

Further transcription factors, including TWIST, ZEB, MEF2A, and FOXC2, stabilize EMT through transcriptional, epigenetic, and metabolic mechanisms. In bladder carcinoma, TWIST expression was higher in muscle-invasive tumors (p = 0.0001), and SNAIL and SLUG expression correlated with muscle invasion (p = 0.005) (Khan et al., 2025). In pancreatic adenocarcinoma, Twist overexpression decreased E-cadherin, increased N-cadherin, Snail, and ZEB1, suppressed CD8^+^ T cell, NK cell, and monocyte infiltration, and established an immunosuppressive microenvironment, correlating with poor overall survival (Li Q. et al., 2024). In metastatic gastric cancer, TWIST-positive circulating tumor cells (CTCs) were detected in 77.4% of patients, and counts exceeding 2.5 per 7.5 mL blood showed a trend toward reduced survival (p = 0.105) (Jhi et al., 2021).

Mechanistic studies have further elucidated the hierarchical and cooperative regulation among EMT-TFs. Ets1 and Ets2 together regulated Snail and ZEB1/2, and dual silencing suppressed TGF-β/Ras-induced Snail expression, with the p54-Ets1 isoform containing exon VII being essential for this regulation (Ichikawa et al., 2022). ZEB1 promoted EMT in lung adenocarcinoma by enhancing endosomal trafficking and plasma membrane protein turnover, establishing polarity and motility (Banerjee et al., 2021). MEF2A directly increased ZEB2 and CTNNB1 to cause EMT, Wnt/β-catenin signaling, tumorigenicity and metastasis in colorectal cancer (Xiao et al., 2021). ZEB2 also interacted with Twist1, PRMT5 and the NuRD complex to inhibit E-cadherin epigenetically, which also strengthened EMT and metastatic capabilities further (Zheng et al., 2022). FOXC2, which is mostly inactive in adult tissues, acted as a transcriptional regulator of FABP4 in breast cancer, facilitating lipid uptake, formation of CSCs, and metastasis (Demestichas et al., 2024). These studies indicate the coordinated network of EMT transcription factors to induce transcriptional programs, epigenetic programs, and metabolic programs to promote tumor progression. Combined, these studies suggest that EMT transcription factors cannot be considered as independent regulators, but rather as a context-dependent and cooperative network, which combines oxidative stress, stromal signaling, immune remodeling, and metabolic adaptation. One common theme found across all tumor types is that SNAIL, SLUG, TWIST, and ZEB proteins do not just invoke loss of epithelial identity; these proteins stabilize plastic intermediate forms that couple invasion with stemness, immune evasion and therapy resistance. Taking this broader view is significant as it transforms EMT-TFs into descriptive biomarkers of aggressive disease into nodes that are mechanistically relevant, and possibly, explain why specific upstream insults focus on similar metastatic phenotypes.

### 3.2 Signaling pathways governing EMT

EMT is controlled by a network of signaling cascades, among which the Hippo pathway and its downstream effectors Yes-associated protein (YAP) and transcriptional co-activator with PDZ-binding motif (TAZ) have been strongly implicated. Dysregulation of Hippo signaling promotes malignant transformation by interacting with EMT transcription factors and driving tumor progression. In phyllodes tumors (PTs) of the breast, nuclear immunopositivity of YAP, TAZ, and their DNA-binding partner TEAD was markedly increased in high-grade tumors (45 benign, 21 borderline, 20 malignant), correlating with EMT regulators Snail and ZEB, thereby implicating Hippo signaling in EMT-associated tumorigenesis (Akrida et al., 2024). In CRC, YAP was significantly upregulated at both mRNA and protein levels in tumor samples compared with adjacent tissues, and its knockdown suppressed proliferation and migration. YAP overexpression enhanced EMT by upregulating vimentin, N-cadherin, and EMT-inducing transcription factors (Snail1, Slug, ZEB1/2), while repressing E-cadherin. Mechanistically, YAP activated the Glut3/AMPK signaling cascade to drive EMT and tumor progression (Jiang et al., 2021). TAZ was significantly overexpressed in tumor tissues and cell lines in pancreatic cancer compared to normal pancreatic epithelium. Functional experiments have shown that TAZ stimulated proliferation, invasion and EMT in the presence of TEAD transcription factors, which was facilitated by silencing the upstream regulator Merlin (Xie et al., 2015). Likewise, the cooperation of YAP and syndecan-2 (SDC2) in facilitating EMT-dependent invasion, metastasis and resistance to drug has been observed in CRC. YAP was found to be highly expressed in 13/16 and SDC2 in 12/16 patients with CRC on analysis of 16 CRC patient specimens. The YAPSDC2 axis mediating EMT by BMP4, CTGF and FOXM1, and YAP overexpression silencing upstream Hippo kinase LATS1 and MST1/2 were demonstrated to support malignancy potential by functional assays (Yang et al., 2023).

Other oncogenic pathways are also related to the EMT regulation and therapeutic resistance besides Hippo signaling. The stemness factor OCT4 had high expression in tumor tissues and ovarian metastases of ovarian cancer and induced EMT by activating PI3K/AKT/mTOR. Silencing of OCT4 inhibited proliferation, invasion and tumorigenicity both *in vitro* and *in vivo* whereas an AKT agonist (SC79) reversed these silencing effects and these effects were directly related to OCT4–AKT interaction leading to EMT (Xie et al., 2022). The complexity of EMT regulation, which is further highlighted by tumor-stromal interaction in CRC, was that a subpopulation of THBS2+ cancer-associated fibroblasts (CAFs) could mediate oxaliplatin resistance via EMT. Multi-omics showed that THBS2 + CAFs secreted COL8A1, which bound to the tumor cells via the ITGB1 receptor, triggered the PI3K/AKT pathway and EMT. High level of COL8A1 increased resistance, whereas ITGB1 knockout or AKT inhibition reverted EMT and sensitivity to chemotherapy were restored (Zhou et al., 2024).

Another quantifiable mechanistic aspect that has definite pharmacological implications is the cross-talk between EMT and ferroptosis. Recent studies also suggest that EMT not only facilitates invasion and resistance to therapy, but also remodels the sensitivity to ferroptosis by integrating alterations in the membrane lipid content, redox buffering, and Hippo-related signaling (Zou et al., 2019). Mesenchymal or p-EMT conditions, especially mediated by ZEB1, lead to the accumulation of phospholipids that contain polyunsaturated fatty acids (PUFA-PLs), preferential substrates in iron-dependent lipid peroxidation (Kagan et al., 2017). Such modification is mediated by the switched expression of lipogenic and lipid-remodeling enzymes, such as ACSL4, ELOVL5, and FADS2 and the corresponding relative inhibition of monounsaturated fatty acid-producing enzymes, such as SCD1, which decreases the ferroptotic death threshold (Viswanathan et al., 2017). Simultaneously, E-cadherin degradation in EMT disrupts the cadherin-NF2/Merlin-Hippo signaling pathway, which allows activation of YAP/TAZ and the expression of ferroptosis-related mediators, including ACSL4, transferrin receptor, EMP1, and NOX family oxidases (Wu et al., 2019). Nevertheless, this state of ferroptosis-prone is context-specific, since EMT-high and therapy-resistant cells can as well employ compensatory antioxidant mechanisms, especially, the SLC7A11-GSH-GPX4 axis and FSP1-CoQH2 pathways, to clear lipid hydroperoxides and avoid cell death (Bersuker et al., 2019). Together, these results indicate that EMT generates a therapeutically vulnerable condition of ferroptosis vulnerability and adaptive protection, making the combination of ferroptosis inducers and inhibitors of GPX4, system xc, SCD1, or other lipid-protective responses a seriously promising approach to tumors enriched with EMT support.

Other EMT-regulating cascades denoted in Figure 1, such as Wnt/β-catenin and NF-kB-signaling, have specific pharmacological significance as both of them combine peripheral cues of inflammation and survival with transcriptional EMT programs. Aberrant Wnt/β-catenin activation stabilizes β-catenin and translocate it into the nucleus, and boosts transcription of EMT-related regulators like ZEB and Snail and matrix-remodeling genes, which maintains invasion, stemness, and metastatic progression (Reya and Clevers, 2005). This signaling can also seem therapeutically amenable, as in breast cancer models chlorogenic acid was shown to directly engage with LRP6, inhibit downstream β-catenin signaling, reestablish epithelial markers including E-cadherin and ZO-1, and downregulate ZEB1, Snail, Slug, MMP-2, and MMP-9, all of which diminish EMT and invasiveness (Xue et al., 2023). Simultaneously, NF-kB plays a central role as an inflammatory EMT amplifier following cytokine, growth factor, and stress signaling where Ikβα phosphorylation enhances nuclear activity of p65 and collaborates with TGF-β/Smad signal relay to stabilize mesenchymal transition (Julien et al., 2007). An exemplary pharmacologic instance is berberine, which in colon cancer interfered with TGF-β/Smad-NF-kB cross-signaling, lowered p-Smad2/3, NF-kB p65 and Ikβα signaling, altered miR-1269a and inhibited the expression of mesenchymal markers (Huang et al., 2024). These illustrations, in combination with the preceding, support the idea that the signaling nodes illustrated in Figure 1 are not only descriptive but are hardened with actionable intervention data, which support Wnt/β-catenin and NF-kB as mechanometrically validated, pharmacologically targetable EMT pathways.

Besides these major findings, various studies have also elucidated the roles of diverse signaling cascades (such as TGF-β/Smad, Wnt/β-catenin, Notch, Hedgehog, MAPK, and PI3K/AKT) in the induction of EMT and tumor progression in various cancer models. Rather than reiterating individual study details in the text, Table 1 integrates the major pathway-level regulators, experimental models, and EMT-associated mechanisms reported in recent literature.

**TABLE 1:** Key signaling pathways and molecular regulators driving EMT across different cancer types.

Cancer type	Experimental model	Regulator/Complex	Pathway	EMT-related mechanism	References
Ovarian cancer	OC cell lines; patient tissues	RGS3	TGF-β/Smad	RGS3 interacts with ARID3B and facilitates SMAD2/3 phosphorylation, promoting EMT, proliferation, migration, and invasion; silencing induces apoptosis	Wang et al. (2025)
Triple-negative breast cancer	TNBC cell lines; patient tissues	CD151 with Integrin α3β1	MAPK	CD151 forms a complex with integrin α3β1 to activate MAPK signaling, enhancing EMT, migration, and invasion; knockdown reduces aggressiveness	Lv et al. (2025)
Colorectal cancer	HCT-116 cells; nude mouse xenograft	SNHG12 (lncRNA)	TGF-β/Smad2/3	SNHG12 regulates TGF-β/Smad2/3 signaling to promote proliferation, migration, invasion, and EMT; TGF-β overexpression rescues knockdown effects	Zhao et al. (2025)
Colorectal cancer	*In vitro* CRC cell lines; nude mouse xenograft; TCGA dataset	FOXC1	TGF-β/Smad2/3 via Snail and CXCL2 loop	FOXC1 upregulation activates Snail-mediated EMT and recruits M2 macrophages through CXCL2, reinforcing TGF-β signaling and sustaining metastasis	Zhang et al. (2025a)
Hepatocellular carcinoma	HCCLM3, Hep3B cells; *in vivo* xenograft; GSEA analysis	Piezo1 and Rab5c	TGF-β	Piezo1 recruits Rab5c to activate TGF-β signaling, enhancing EMT, proliferation, invasion, and metastasis	Li et al. (2022b)
Glioblastoma	U87, U251 cells; nude mouse xenograft	MICAL2 with TGFRI	TGF-β/p-Smad2	MICAL2 interacts with TGFRI to activate TGF-β/p-Smad2 signaling, driving EMT-like changes, proliferation, invasion, and tumor growth	Pu et al. (2021)
Pan-cancer	RNA-seq, RIP, ChIP; xenograft; nanoparticle delivery	lncRNA Smyca with Smad3/4 and c-Myc/Max	TGF-β/Smad and c-Myc	Smyca scaffolds Smad3/4 and c-Myc/Max, amplifying TGF-β and c-Myc signaling to promote EMT, metabolic reprogramming, invasion, stemness, and chemoresistance	Chen et al. (2022)
Pancreatic cancer	*In vitro* PC cell lines; TCGA and GEO datasets; tissue arrays	CAPN2 (Calpain-2)	Wnt/β-catenin	CAPN2 overexpression activates Wnt/β-catenin signaling, promoting EMT, proliferation, invasion, and migration; knockdown suppresses progression	Peng et al. (2022)
Hepatocellular carcinoma	TCGA and GEO datasets; RT-qPCR and IHC validation; HCC cell lines	SMG9	Wnt/β-catenin	SMG9 overexpression activates Wnt/β-catenin signaling, driving EMT, proliferation, invasion, and apoptosis resistance	Jin et al. (2021)
Pancreatic ductal adenocarcinoma	PSCs with PITX2 knockdown; co-culture with PC cells; tissue microarray	PITX2 in pancreatic stellate cells	Wnt/β-catenin	PITX2 promotes PSC proliferation and induces EMT in PC cells via β-catenin stabilization; knockdown suppresses invasion and migration	Wu et al. (2023a)
Lung adenocarcinoma	Lung cancer cell lines; mouse xenograft models	HORMAD1	PI3K/AKT/GSK-3β/Wnt/β-catenin	HORMAD1 enhances AKT and GSK-3β phosphorylation, stabilizing β-catenin to drive EMT, proliferation, and metastasis	Liu et al. (2022a)
Hepatocellular carcinoma	HCC cell lines; xenograft models	IGFBP4 (regulated by MYBBP1A)	MYBBP1A/IGFBP4/NOTCH1 axis	IGFBP4 downregulation (via MYBBP1A-driven hypermethylation) activates NOTCH1 signaling and EMT; IGFBP4 restoration suppresses metastasis	Sun et al. (2025b)
Triple-negative breast cancer	TNBC cell lines; xenograft mouse models	PKMYT1	Notch	PKMYT1 promotes proliferation, migration, invasion, and EMT through Notch activation; knockdown reduces metastasis	Li et al. (2024a)
Glioma	Glioma cell lines; xenograft mouse model; patient samples	FAM3C	Notch	FAM3C promotes proliferation, invasion, EMT, and survival through Notch signaling; knockdown or Notch inhibition suppresses tumor progression	Xia et al. (2024)
Oral squamous cell carcinoma	OSCC cell lines; xenograft models; TCGA dataset	Gli2 (Hedgehog TF)	Hedgehog and Wnt/β-catenin	Gli2 activation drives EMT, invasion, and proliferation; knockdown inhibits malignant progression	Wang et al. (2024b)
Pancreatic cancer	PANC-1 and BxPc-3 cells; knockdown and overexpression assays	Smad4 and Gli1	TGF-β/Smad and Hedgehog-Gli axis	TGF-β1 induces EMT via Smad2/3; Hedgehog-Gli1 mediates downstream activation; Smad4 suppresses Gli1, regulating EMT and invasion	Guo et al. (2024)

### 3.3 Tumor microenvironment as a driver of EMT

The TME is an active and multifaceted ecosystem that actively orchestrates epithelial -mesenchymal transition (EMT) by coordinating immune, stromal, and molecular interactions. Immune -regulatory processes have the central role in creating this pro-EMT environment. MiR 192-5p has been demonstrated to mediate EMT and proliferation in gastric cancer by regulating RB1 and NF-κBp65, which subsequently enhanced IL-10 secretion and regulatory T cell (Treg) differentiation into an immunosuppressive feedback loop that strengthened EMT and tumour progression (Song et al., 2022). A pan-cancer analysis revealed that EMT-high tumors consistently exhibited enrichment of tumor-associated macrophages (TAMs), exhausted CD8^+^ T cells, and immunosuppressive cytokines such as TGFB1 and IL10, confirming that immune modulation sustains EMT across cancer types (Tiwari et al., 2022). Similarly, in TNBC, restoration of miR-200c reversed EMT and reactivated M1 macrophage polarization through GM-CSF upregulation, indicating that EMT-driven immunosuppression is reversible through cytokine reprogramming (Williams et al., 2021). Cytokine signaling also directly induces EMT; IL-1β stimulated p-EMT in lung epithelial cells via coordinated activation of PI3K/AKT and MEK/ERK cascades downstream of EGFR and IL-1R (Tabei and Nakajima, 2024), whereas IL-17A enhanced EMT and invasion in lung adenocarcinoma by activating the NLRP3 inflammasome and IL-1β secretion (Liu W. et al., 2022).

Beyond immunoregulation, hypoxia and metabolic stress profoundly influence EMT through adaptive signaling pathways. In GC, hypoxia-induced lncRNA HCP5 acted as a competing endogenous RNA for miR-186-5p, upregulating WNT5A and promoting EMT, invasion, and proliferation (Gao et al., 2021). Similarly, TGF-β1 signaling under hypoxic conditions triggered autophagy in fibroblasts, transforming them into cancer-associated fibroblasts (CAFs) with increased glycolytic flux and MCT4 expression, thereby fueling EMT via lactate exchange with tumor cells (Jena et al., 2022). Furthermore, a genome-scale CRC analysis revealed that high TGFBscore tumors had activated EMT, immunosuppressive cell infiltration, and metastatic potential, which supported TGF-β as a master regulator of EMT via TME remodeling (Tao et al., 2024).

Another important stimulator of EMT in the TME is fibroblast-mediated remodeling of the extracellular matrix (ECM). A unique subpopulation of fibroblasts that exhibits ECM remodeling activity facilitated the progression of adenoma to carcinoma through the communication activity of COL1A2 in gallbladder cancer through the linkage of stromal dynamics and EMT activation (Ma et al., 2025). PDGFR-2/Cav-1 signal transduction in CAFs stimulated autophagy via the mTOR/FIP200/ATG13 pathway, promoted glycolysis and lactate export via HIF-1/MCT4/MCT1 in tumor cells, promoting EMT and migration (Zhang L. et al., 2025). Another study, single-cell transcriptomics of oral squamous cell carcinoma (OSCC), found that strong ligand-receptor interactions (TNFSF12–TNFRSF25/TNFRSF12A) between CAFs and p-EMT tumor cells exist, and these interactions form a signaling circuit that maintains EMT-like phenotypes (Huynh et al., 2022).

Although the TME is a significant trigger of EMT, growing evidence implicates that EMT, conversely, reciprocally reorganizes the TME to create a feedback loop that strengthens the evolution of the tumor. The TME actively is remodelled by EMT-mediated tumor-stromal and exosomal signaling. In CRC, myeloid-derived suppressor cells (MDSCs), TAMs, and Tregs were recruited in a stage-dependent manner through EMT-associated invasive tumor programs (EMTPs), and inflammatory niches were replaced by hypoxic ones as the tumor was evolving (Wang et al., 2023). Intermediate EMT conditions in breast cancer were found to restructure the TME through immigration of immune cells with pro-tumorigenic phenotypes and distinct epigenetic signatures (Lu et al., 2024). Exosome-mediated signaling further amplifies this crosstalk; TNBC-derived exosomes enriched in MMP-1 activated PAR1 in recipient cells, triggering EMT and metastasis, while metastatic cells further secreted MMP-1–rich vesicles to sustain a pro-invasive environment (Zhu et al., 2022). Moreover, SERPINB5 enhanced TNF-α/NF-κB signaling in CRC and stimulated EMT, angiogenesis, endothelial activation, and correlated inflammatory and vascular remodeling with EMT progression (Liu B.-X. et al., 2024).

### 3.4 Epigenetic and non-coding RNA regulation

Epigenetic mechanisms driving EMT is mainly achieved by DNA methylation and histone modifications, which control the tumor progression by dynamically regulating gene expression. DNA methylation sets up transcriptional programs ensuring epithelial integrity or enabling mesenchymal plasticity during stress. Promoter hypermethylation of epithelial adhesion gene CDH1 and EPCAM and hypomethylation of the mesenchymal gene TWIST2 resulted in stable chemoresistance and EMT in cisplatin-resistant ovarian cancer cells (A2780cis). These patterns of methylations were reversed by treatment with the DNA methyltransferase inhibitor 5-azacytidine, which restored epithelial expression of genes and suppressed mesenchymal characteristics (Alghamian et al., 2022). Likewise, promoter-methylation-mediated changes in EMT-related gene expression were caused by hypoxia and inflammatory stimuli in pancreatic ductal adenocarcinoma (PDAC). The level of methylation was decreased by the administration of decitabine as much as 53%, resulting in the reinstatement of silenced EMT-related genes and establishing that the regulation is a methylation-driven mechanism (Urbanova et al., 2022). With bladder cancer, EMT marker Vimentin (VIM) epigenetic regulation showed comparable pattern with promoter methylation and expression level rising according to disease stage, which was associated with reduced E-cadherin levels. These effects were reversed using demethylation or histone deacetylase inhibition, which decreased migration and invasion *in vitro* and *in vivo* (Monteiro-Reis et al., 2023).

Beyond mechanistic research, tumor-centric patterns of DNA methylation have been demonstrated to influence EMT activation and clinical outcomes. Hypermethylation of SPAG6 promoters was a potent downregulator of mRNA expression in all molecular subtypes of breast cancer and overexpression of SPAG6 stimulated EMT by increasing SNAIL, TWIST1, and Vimentin, and decreasing E-cadherin levels. It resulted in a significant increase in migration (p < 0.0001) and colony formation (p = 0.0004), reflecting that SPAG6 suppression through methylation is a contributing factor to tumor aggressiveness (Mijnes et al., 2022). In hepatocellular carcinoma (HCC), methylation of TGFβ target genes mediated the pathway’s switch from tumor suppression to EMT induction. Exposure to Decitabine increased EMT related transcription factors including SNAI1/2 and ZEB1/2 and hypomethylation of the SNAI1 promoter was associated with high grade tumors, metastasis and recurrence (Bévant et al., 2021). In addition, promoter hypermethylation of the tumor suppressor gene PCDH17 by DNMT3B blocked its expression and promoted proliferation, migration and invasion by activating EMT (Liu Y. et al., 2022).

The histone modifications work in conjunction with DNA methylation to control EMT through chromatin structure and transcriptional activity. In pancreatic cancer, hypoxia-induced NADPH oxidase 4 (NOX4) inactivated lysine demethylase 5A (KDM5A), leading to increased H3K4 trimethylation (H3K4me3) at the SNAIL1 promoter, thereby enhancing transcription and promoting invasion and metastasis. NOX4 deficiency suppressed hypoxia-induced EMT, while KDM5A knockdown restored H3K4me3 levels, confirming the NOX4–KDM5A–H3K4me3 axis as a critical regulator (Li H. et al., 2021). KDM2B similarly mediated TGF-β-induced EMT in lung and pancreatic cancer cells via H2AK119 monoubiquitination within Polycomb Repressive Complex 1 (PRC1), recruiting EZH2 and inducing H3K27 methylation at epithelial gene loci, resulting in transcriptional repression and EMT promotion (Wanna-Udom et al., 2021). In breast cancer, Ring1b, a PRC1 subunit, formed complexes with DEAD-box RNA helicases and EMT transcription factors on the E-cadherin promoter, inducing H2A monoubiquitination, repressing CDH1, and driving partial-to-full EMT. High Ring1b expression correlated with metastasis and poor prognosis (Wang et al., 2021c).

Histone acetylation and lactylation integrate extracellular and microenvironmental cues to further modulate EMT. In gallbladder cancer, increased matrix stiffness activated fibroblasts to secrete SEMA7A, which engaged integrin β1 on cancer cells and activated AKT signaling, resulting in phosphorylation of the histone acetyltransferase p300 at Ser1834. This enhanced histone acetylation and transcription of EMT genes, promoting migration, invasion, and stem-like properties *in vitro* and *in vivo* (Sun L. et al., 2025). In head and neck squamous cell carcinoma, hypoxia-induced H3K4 acetylation (H3K4Ac) at CDH1 and VIM promoters, regulated by HDAC3 and WDR5, facilitated migration and invasion, with GLI1 and SMO identified as novel H3K4Ac-marked EMT genes correlating with metastasis (Wang J. Q. et al., 2020). SMYD2 overexpression in lung cancer promoted EMT-associated migration, invasion, and metastasis via SMAD3 regulation, while knockdown suppressed these processes (Kim et al., 2023). Furthermore, in gastric cancer, cancer-associated fibroblasts induced lactate-mediated H3K18 lactylation (H3K18la) at the PD-L1 promoter via the LOX/TGFβ/IGF1 axis, increasing transcription and potentially reducing PD-1/PD-L1 blockade efficacy (Li Z. et al., 2024).

Collectively, these findings highlight those epigenetic alterations, including DNA methylation, histone modifications, and chromatin remodeling, intricately modulate EMT progression across various cancers. In addition to these classical mechanisms, ncRNAs have become a critical post-transcriptional regulator that interacts synergistically with epigenetic modifiers to tune EMT-related gene expression. Overall, these results point to the conclusion that ncRNAs are multifaceted post-transcriptional modulators of EMT by regulating EMT transcription factors, signaling intermediates, and chromatin-connected programs. To prevent the repetition of separate ncRNA instances in the narrative, Table 2 provides an overview of representative ncRNA groups, their main EMT targets, and their pathophysiological implications in cancer types. The therapeutic implication of this mechanistic convergence is significant: EMT-directed intervention cannot be expected to be effective on a single target, but rather it has to involve pathway-sensitive and state-sensitive interventions, to disrupt plasticity, metastatic competence and adaptive resistance. Based on this, the next section will explain current therapeutic strategies to target EMT using epigenetic, RNA-based, immune as well as pharmacological methods.

**TABLE 2:** Role of non-coding RNAs in EMT gene regulation across different cancer types.

Non-coding RNA	Cancer/Cell type	Target gene/EMT TF	EMT regulation and functional effect	References
miR-21	Cervical cancer (HeLa, SiHa; patient tissues)	ZEB1	Upregulates ZEB1, increases Vimentin and N-cadherin, decreases E-cadherin, promoting EMT, migration, and invasion	Tang et al. (2020b)
miR-9-3p	Breast cancer (Gem-treated cell lines)	MTDH	Downregulates MTDH, regulating EMT markers (E-cadherin, N-cadherin, Vimentin), reducing migration and invasion	Wang et al. (2021b)
miR-9-5p	Renal cell carcinoma (cell lines and tumor tissue)	EMBP1	Increases epithelial markers (E-cadherin, claudin) and decreases Vimentin, inhibiting EMT, proliferation, and migration	Hong et al. (2020)
miR-9-5p	Hepatocellular carcinoma (cell lines)	CPEB3	Downregulates CPEB3, elevates Vimentin, reduces E-cadherin, promoting EMT, proliferation, and invasion	Shu et al. (2021)
miR-92a-3p	Cervical cancer stem cells	LATS1	Downregulates LATS1, increases Vimentin and TAZ, decreases E-cadherin, promoting EMT and invasion	Liu et al. (2021a)
lncRNA LETS1	Breast and lung cancer cells (*in vitro*)	TβRI/SMAD2/3/SMAD7	Activates SMAD2/3 signaling and inhibits SMAD7 via NR4A1 induction, promoting EMT and migration	Fan et al. (2023)
lncRNA PVT1	Ovarian cancer (SKOV3, CAOV3 cell lines)	CTGF/E-cadherin/Vimentin	Upregulates CTGF and Vimentin, downregulates E-cadherin, enhancing EMT and migration	Dong et al. (2023)
lncRNA SNHG1/miR-15b	Gastric cancer (cell lines)	DCLK1/Notch1	SNHG1 enhances DCLK1 and Notch1 expression, promoting EMT, migration, and invasion; miR-15b suppresses EMT via DCLK1 inhibition	Liu et al. (2020)
lncRNA HOTAIR/miR-145-5p	Liver cancer (HepG2, SNU-387; patient tissues)	NUAK1/PRC2 (EZH2)	HOTAIR represses miR-145-5p, upregulates NUAK1, and activates EMT through epigenetic regulation	Chu et al. (2023)
lncRNA NEAT1/miR-361-3p	Non-small cell lung cancer (NSCLC)	HMGA1/METTL3	METTL3-mediated m6A modification stabilizes NEAT1, which sponges miR-361-3p to elevate HMGA1, promoting EMT and proliferation	Qi et al. (2023)
lncRNA CASC15/miR-23b-3p/miR-24-3p	Ovarian cancer	SMAD3	CASC15 sponges miR-23b-3p/miR-24-3p to upregulate SMAD3, promoting TGF-β-induced EMT and metastasis	Lin et al. (2022)
circ-10720 (Cullin2 circular RNA)	Hepatocellular carcinoma (HCC)	Vimentin/Twist1	Acts as a sponge for miRNAs targeting Vimentin; enhances Twist1-induced EMT and tumor progression	Meng et al. (2018)
circITGB6	Colorectal cancer (CRC)	PDPN/TGFβ pathway	Stabilizes PDPN mRNA via IGF2BP3 binding, activating TGFβ-mediated EMT and promoting metastasis	Li et al. (2023b)
hsa_circ_0008726	Pre-eclampsia (HTR-8/SVneo trophoblast cells)	miR-345-3p/RYBP	Sponges miR-345-3p to increase RYBP expression; regulates trophoblast EMT, migration, and invasion	Shu et al. (2022)
circ_000999	Lung cancer (LC; human bronchial epithelial cells)	miR-205-5p/ZEB1	Acts as a sponge for miR-205-5p, releasing ZEB1 repression and promoting EMT and migration	Wang et al. (2024a)
circCSPP1	Colorectal cancer (CRC)	miR-193a-5p/COL1A1	Sponges miR-193a-5p to enhance COL1A1 expression, promoting EMT, migration, and invasion	Wang et al. (2020b)
piR-17560 (exosomal piRNA-17560)	Breast cancer	FTO/ZEB1/YTHDF2	Increases FTO-mediated ZEB1 mRNA stability by reducing m6A methylation, promoting EMT and chemoresistance	Ou et al. (2022)
SNORA71A	Breast cancer (MCF-7, MDA-MB-231)	ROCK2/G3BP1	Promotes EMT via G3BP1-mediated ROCK2 stabilization; enhances migration and metastasis	Hu et al. (2022)

## 4 Therapeutic horizons targeting EMT

### 4.1 Epigenetic and RNA-based therapeutics

Epigenetic modulation has emerged as a promising approach to reverse EMT and inhibit cancer metastasis. Histone deacetylase inhibitors (HDACis) have been found to reverse EMT by recovering epithelial phenotypes. Treatment of MCF-7 cells with the HDAC inhibitor Trichostatin A (TSA) in breast cancer led to a significant decrease in invasion and migration because it increased the protein epithelial marker E-cadherin and decreased the protein mesenchymal marker vimentin and the EMT-inducing transcription factor SLUG (SNAI2). SLUG overexpression stimulated EMT, and TSA counteracted them, indicating that it can inhibit SLUG-mediated transcriptional regulation of EMT (Wang X. et al., 2020). Likewise, GSK-J4 inhibited JMJD3/KDM6B in prostate cancer, which restored the H3K27me3 level, suppressing the activity of mesenchymal genes and inhibiting TGF-β-mediated EMT (Dalpatraj et al., 2023). Consistent with these findings, HDAC1, HDAC3 was also observed to cause epigenetic silencing of Snail2 transcription in liver cells via deacetylation of H3K4 and H3K56 histone residues in response to TGF-β-mediated EM. This process inhibited the EMT-related migration and invasion *in vitro* and *in vivo*, which proves that particular histone-modifying enzymes may serve as negative modulators of EMT, and may be used as a therapeutic agent to prevent metastasis (Hu et al., 2020).

Complementing these findings, RNA-based therapeutics have demonstrated significant potential in modulating EMT-pathways in a variety of different cancers. In gastric cancer, lnc-CTSLP4 was markedly downregulated, and its restoration inhibited EMT and peritoneal dissemination by binding the Hsp90α/HNRNPAB complex and recruiting the E3-ubiquitin ligase ZFP91, leading to HNRNPAB degradation and suppression of Snail transcription (Pan et al., 2021). Similarly, in glioblastoma, downregulated LINC-PINT inhibited EMT by blocking Wnt/β-catenin signaling, thereby reducing tumor proliferation and invasion (Zhu et al., 2021). In ovarian cancer, the tumor-suppressive piRNA piR-26681 enhanced the stability of METTL3/METTL14 and promoted m6A methylation, which stabilized METTL14 and FBXO16 mRNAs, downregulating β-catenin and inhibiting WNT/EMT signaling (Chen et al., 2024). In prostate cancer, miR-145-5p was shown to inhibit EMT and bone metastasis by suppressing bFGF, IGF, and TGF-β signaling, increasing E-cadherin, and reducing MMP-2 and MMP-9 expression (Luo B. et al., 2022). Likewise, in hepatocellular carcinoma, miR-125b-5p suppressed EMT and cancer stemness by targeting STAT3 and inhibiting Wnt/β-catenin signaling; its delivery via a folate-conjugated nanocarrier allowed real-time MRI-based therapeutic monitoring, offering a precision nanomedicine approach for EMT-targeted therapy (Guo et al., 2019).

Further examples of clinical potential of RNA-based EMT modulation are the use of small RNA interference and antisense oligonucleotide (ASO) technologies. Exosome-mediated delivery of TRPP2 siRNA in head and neck cancer reversed EMT and metastatic properties by suppressing TRPP2 expression, stimulating E-cadherin, and suppressing N-cadherin and vimentin expression (Wang et al., 2019). The mesoporous silica nanoplatform that was loaded with PDLIM5 siRNA restored the sensitivity of gefitinib in NSCLC by inhibiting the TGF-β-induced EMT and overcoming resistance to EGFR-TKI (Wu H. et al., 2023). STAU2, an RNA-binding protein in pancreatic ductal adenocarcinoma, facilitated EMT and metastasis through Palladin (PALLD), a cytoskeletal-associated protein; antisense oligonucleotide (STAU2-ASO)-mediated inhibition of STAU2 was effective *in vivo* in suppressing these downstream targets and the progression of the tumors (Ding J. et al., 2025).

### 4.2 Immunotherapy and EMT

**FIGURE 2:**
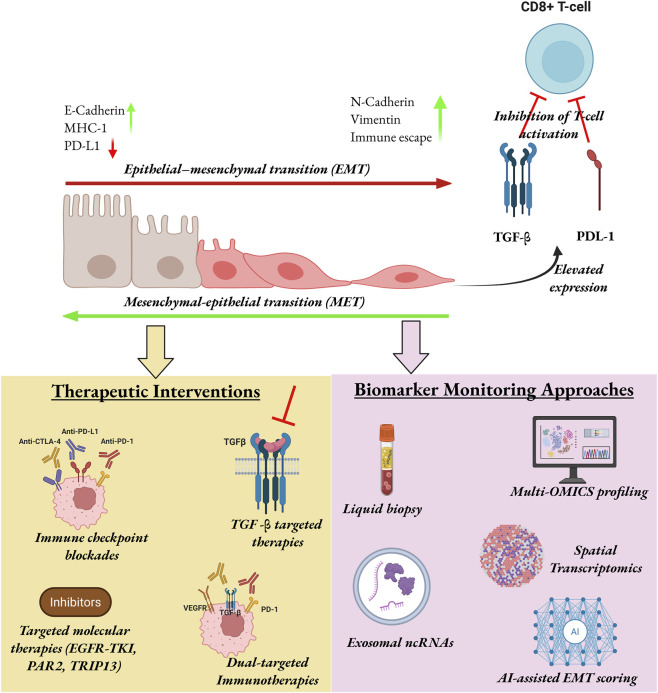
EMT plasticity and translational control in cancer, illustrating the shift from epithelial to mesenchymal states together with associated changes in immune escape and therapeutic vulnerability. The lower panels outline the precision therapeutic strategies (left) that includes immune checkpoint blockade, TGF-β-targeted therapy, dual-target immunotherapy, targeted molecular therapies; and biomarker monitoring approaches (right) that includes liquid biopsy, multi-omics profiling, spatial transcriptomics, exosomal ncRNAs, and AI-assisted EMT scoring. Image created with BioRender.com.

EMT has gradually been identified as a critical contributor to tumor immune evasion, not only as an essential mediator of metastasis but as a regulator of immune suppression as illustrated in Figure 2. Instead of facilitating cellular plasticity, EMT physically reconfigures the TME to an immunoevasive state. An example is breast cancer, where EMT was discovered to confer decreased immunogenicity and increased immune tolerance to tumor cells, with the reactivation of this program exposing new neoantigens produced by EMT-regulated gene expression and alternative splicing, increasing immune recognition (Camp et al., 2022). Beyond a single cancer model, a pan-cancer study of 17 solid malignancies has revealed that EMT activation is associated with immune infiltration changes, chromosomal instability and expression of checkpoint molecules, which have all been shown to have joint impacts on clinical outcomes and immunotherapy response (Wang G. et al., 2021). A combination of these findings highlights that EMT is not only a structural change but a functional state that defines the immune landscape in cancers.

Following this insight, several studies have investigated the role of EMT in contributing to therapy resistance when interacted with immune checkpoint signaling. Therapy with EGFR-tyrosine kinase inhibitors (EGFR-TKIs) in NSCLC was observed to cause the EMT, which created a self-stimulating feedback loop that undermined the sensitivity of drugs and maintained immune escape (Cruz et al., 2025). Similar mechanisms were observed in HCC, where Sorafenib treatment induced TGF-β1-mediated EMT along with PD-L1 upregulation. TGF-β inhibition by SB431542 lowered the expression of PD-L1, and PD-L1 knockdown decreased EMT, and *vice versa*, showing a two-way regulatory linkage between EMT and immune checkpoints (Shrestha et al., 2021). This molecular pathway-immune suppression cross-linkage was further demonstrated in breast cancer, in which silencing of the long non-coding RNA NEAT1 by using an LDH@si-NEAT1 delivery system inhibited EMT simultaneously with reprogramming tumor-associated macrophages to M2 to M1 and promoting responsiveness to anti-PD-1 therapy via miR-1413p-mediated targeting of PD-L1 (Xiao et al., 2025).

Building on these findings, more recent studies have addressed pharmacological and biologic drugs, which combine EMT inhibition with immune activation. In colon cancer, TRIP13 was inhibited by the small-molecule inhibitor DCZ0415 reducing EMT by suppressing FGFR4, STAT3, and NF-kB signaling, as well as reducing PD-1 and CTLA-4 expression and increasing cytotoxic effectors like granzyme B and perforin (Agarwal et al., 2022). Likewise, the immunomodulatory potential of the naturally occurring melatonin was demonstrated to reduce EMT and PD-L1 expression in head and neck squamous cell carcinoma (HNSCC) through the ERK1/2/FOSL1 pathway, which resulted in greater infiltration of CD8 + T-cells and enhanced the efficacy of the anti-PD-1 therapy (Luo X. et al., 2022). In NSCLC, resistance to osimertinib was also linked to EMT-associated PD-L1 upregulation, where blockade of PAR2 disrupted ERK signaling, reversed EMT, and enhanced sensitivity to EGFR-TKI therapy (Jiang et al., 2022). In addition to these small molecule and RNA based solutions, there is the case of dual-targeting biologics that have shown impressive potential. The multi-potent TGF-β-VEGF antagonist Y332D was capable of reversing EMT and increasing lymphocyte infiltration promoting the effects of PD-1 blockade (Niu et al., 2023). In the same manner, TGF-β and PD-L1 bispecific antibody YM101 reversed immunotherapy resistance by reversing EMT-induced immunosuppression, and its effects were enhanced by TGF-β agonist Mn^2+^, which facilitated dendritic cell activation and T-cell priming (Yi et al., 2021).

### 4.3 Natural compounds and functional foods against EMT

Signaling pathways and transcriptional regulators interact dynamically in EMT, creating numerous nodes of therapeutic interest, which make it an ideal anticancer therapy target. The natural compounds and functional foods have become effective modulators of these networks that revert the epithelial properties and inhibit the aggressiveness of the tumors. In breast cancer, a topoisomerase, Teniposide, reverses EMT by silencing transcription factor, ZEB2, in response to IRF7NMI by inhibiting regulation of mesenchymal-like invasiveness by impairing RNA polymerase I activity and rRNA biogenesis (Metge et al., 2024). In ovarian cancer, cinnamaldehyde, derived as a bioactive compound of cinnamon bark, suppresses PI3K/AKT/mTOR signaling caused by EGF, decreases the pathway components phosphorylation, inhibits proliferation, migration, and invasion, and induces apoptosis through cleaved-PARP and cleaved-caspase 3, and suppresses EMT induction by EGFs through AKT activators *in vitro* and *in vivo* (Wang et al., 2022). Likewise, matrine, an alkaloid of *Sophora flavescens*, in colon cancer targets EMT and vasculogenic mimicry through Claudin-9 downregulation and inhibition of JNK and ERK phosphorylation disrupting actin filament integrity, migration, and invasion and promoting cellular apoptosis (Du et al., 2023). In pancreatic ductal adenocarcinoma, cannabidiol (CBD), a non-psychoactive phytocannabinoid, suppresses EMT by inhibiting MALAT1-mediated activation of the PI3K/AKT/mTOR signaling pathway, reversing CXCL12-induced mesenchymal phenotypes and restoring epithelial markers, while synergizing with gemcitabine to reduce tumor progression without cytotoxicity (Kim et al., 2025). Natural compounds and functional foods regulate EMT by using few recurrent mechanistic cores such as TGF-β/Smad, PI3K/AKT, Wnt/β-catenin, oxidative stress signaling, and epigenetic modulators. To maintain the primary text itself devoted to these more overarching patterns of pharmacology, instead of more or less repeated specifics of the compounds, Table 3 reports some representative compounds, their tumor models, EMT targets, and their main mechanisms of action. In these works, the focus of natural compounds keeps falling to a select few EMT-relevant targets, especially PI3K/AKT/mTOR, Wnt/β-catenin, inflammatory signaling and oxidative stress pathways, and EMT-related transcription factors. This trend indicates that, regardless of chemical diversity, numerous natural actors are acting by destabilizing the signaling plasticity underlying EMT plasticity. The rest of the representative compounds and molecular mechanisms are summarized in Table 3.

**TABLE 3:** Studies investigating natural compounds and functional foods targeting EMT.

Compound/Functional food	Cancer type	EMT markers/Targets	Mechanism of EMT inhibition	References
Montelukast	Triple-negative breast cancer	E-cadherin, Vimentin, SIRT1, VEGF, AKT	Reverses mesenchymal phenotype by downregulating SIRT1, decreases AKT phosphorylation, upregulates E-cadherin, inhibits angiogenesis	El-Ashmawy et al. (2023)
Fatostatin	Glioblastoma multiforme	Vimentin, Snail, GPX4	Suppresses EMT via inhibition of AKT/mTORC1 signaling, induces ferroptosis through AKT/mTORC1/GPX4 axis; enhanced delivery via p28-functionalized PLGA nanoparticles	Cai et al. (2023)
EGCG/Green tea extract	Cervical cancer	Vimentin, ZEB, Slug, Snail, Twist, E-cadherin	Inhibits TGF-β-induced ROS generation, blocks Smad2/3 phosphorylation, nuclear translocation, and DNA-binding activity, restores E-cadherin	Ho et al. (2013)
Propolin G	Triple-negative breast cancer	E-cadherin, Vimentin, Snail, α-tubulin	Activates GSK-3β to promote Snail degradation, inhibits HDAC6 activity to increase vimentin acetylation and degradation	Pai et al. (2022)
Genistein	Hepatocellular carcinoma	miR-1275, EIF5A2, PI3K, Akt	Upregulates miR-1275 to epigenetically inhibit EIF5A2/PI3K/Akt signaling, reduces proliferation, invasion, migration, and stemness	Yang et al. (2022)
Grape Seed Proanthocyanidins (GSPs)	Bladder cancer	E-cadherin, ZO-1, N-cadherin, Vimentin, Slug, MMP-2, MMP-9	Restores epithelial markers, suppresses mesenchymal markers, inhibits phosphorylation of Smad2/3, Akt, Erk, and p38, blocking TGF-β-mediated EMT	Yang et al. (2021)
Sulforaphane	Breast cancer	E-cadherin, N-cadherin, Vimentin, MMP-9, HDAC5	Downregulates HDAC5, reduces mesenchymal markers, restores epithelial phenotype, decreases tumor proliferation and metastasis	Jinfang et al. (2021)
Lycopene	Lung cancer	E-cadherin, N-cadherin, Vimentin, p-PI3K, p-AKT	Inhibits PI3K/AKT signaling, suppressing EMT and metastatic behavior; confirmed via PI3K silencing and overexpression studies	Gu et al. (2025)
Chlorogenic acid	Breast cancer	E-cadherin, ZO-1, ZEB1, N-cadherin, Vimentin, Snail, Slug, MMP-2, MMP-9, LRP6	Directly interacts with LRP6, downregulates LRP6 and β-catenin, suppresses Wnt/β-catenin pathway, inhibits EMT and invasiveness	Xue et al. (2023)
Magnolol	Retinoblastoma	E-cadherin, ZEB1, α-SMA, Fibronectin-1, miR-200c-3p	Upregulates miR-200c-3p to suppress ZEB1, restores epithelial phenotype, reduces migration, invasion, and metastasis	Lai et al. (2023)
Berberine	Breast cancer	E-cadherin, Vimentin, SIRT1, p-AKT, VEGFR2	Reverses mesenchymal phenotype by inhibiting SIRT1/AKT axis, suppresses VEGFR2 signaling and metastasis; enhanced by sirtinol co-treatment	El-Ashmawy et al. (2025)
Berberine	Colon cancer	p-Smad2/3, NF-κB p65, p-IκBα, miR-1269a	Disrupts TGF-β/Smad–NF-κB crosstalk, reduces mesenchymal marker expression, modulates miR-1269a to inhibit EMT	Yang and He (2024)
Oridonin	Nasopharyngeal carcinoma	E-cadherin, Vimentin, Twist1, p-AKT, p-STAT3	Inhibits AKT/STAT3 pathway, restores epithelial phenotype, reduces migration and invasion	Liu et al. (2021b)
Resveratrol	Gastric cancer	E-cadherin, N-cadherin, Vimentin, Snail, Hippo-YAP	Suppresses Hippo-YAP pathway, inhibits TGF-β1-induced EMT, reduces proliferation, migration, and invasion	Deng et al. (2022)

In general, the present EMT-targeted therapeutic options indicate that the most promising are not those disrupting a single EMT inducer, but the ones disrupting EMT-related plasticity networks when combined with immune, metabolic, epigenetic, or traditional anticancer therapies. Not only is the challenge in identifiability of successful EMT modulators, but also whether to select the right modulator to the right EMT state, tumor environment, and resistance phenotype.

## 5 Challenges and future perspectives

There is still a challenge in translating EMT biology in the preclinical discovery into clinically actionable oncology. Despite significant innovations that have illuminated the molecular and cellular underpinnings of EMT, its dynamic, contextual, and reversible attributes remain a barrier to the standardization of biomarkers and their therapeutic use. These obstacles will need to be overcome to transform mechanistic understanding into precision-oncology approaches that are clinically useful.

### 5.1 Limitations of current EMT biomarkers in clinical settings

The dynamic continuum of the EMT is one of the major limitations for the clinical application of EMT biomarkers. The cancer cells shift the EMT phenotypes and can reversibly transition between epithelial, hybrid, and mesenchymal states depending on the state of the TME (Pal et al., 2021). Hence, this EMT plasticity fundamentally poses the challenge to the existing static biomarker assessment strategy.

Another major hurdle is the intratumoral heterogeneity of cancer tissues that limits the identification of EMT biomarkers. Since, the tumor tissues may have epithelial, hybrid, or mesenchymal cell populations, and single tissue biopsies leads to sampling bias and have the high chance of misrepresenting the overall EMT landscape among the heterogeneous tumors (Pal et al., 2021). Although, the liquid biopsy and advanced imaging approaches offers a promising invasive method for monitoring EMT, they are hugely limited by the full spectrum of phenotypic states of EMT. Detection of circulating tumor cells (CTCs) is a strategy for EMT biomarker, however its lacks the complex phenotypic states of EMT (Li D. et al., 2023).

One of the greatest translational obstacles in CTC-based EMT measurement is that CTC heterogeneity is not only biological, but also technological. Numerous clinically relevant enrichment platforms have been based on epithelial proteins like EpCAM or cytokeratins, but the immune response of EMT also frequently silences such antigens, inducing mesenchymal and hybrid epithelial/mesenchymal CTCs to evade capture and resulting in systematic underrepresentation of clinically relevant subpopulations (Lecharpentier et al., 2011). This issue is also further exacerbated by the fact that CTCs have an extremely low abundance and short half-life in peripheral blood, pre-analytic variability in the collection and processing of blood and contamination of conditions by leukocytes or platelet-coated tumor cells, which could obscure proper phenotypic classification (Koch et al., 2020; Hamza et al., 2021). Furthermore, CTC clusters and intermediate EMT can exhibit a mixed morphology and expression of markers, and it is challenging to establish consistent cut-off values across imaging, immuno-staining and transcriptomic modalities. As a result, the variations in enrichment strategy, antibody panel, scoring algorithms, and reporting threshold limit the reproducibility of studies, and limit regulatory-level standardization. New directions in future studies must thus involve marker-agnostic or combined biophysical-biochemical isolation systems, single-cell and spatially resolved multi-omics characterization, serial longitudinal sampling, and bridging CTC analysis to ctDNA and exosomal RNA as well as clinical imaging to better reflect EMT plasticity and enhance clinical translation of CTC-based biomarkers.

Also, the conventional immune-histochemical and molecular assays lack sensitivity in detection of EMT biomarker in either p-EMT or hybrid EMT states. For instance, a recent study reported that the single-cell sequencing revealed that hybrid EMT cells co-express both E-cadherin (epithelial marker) and vimentin (mesenchymal marker) and promotes metastasis therapeutic resistance (Sinha et al., 2020). However, although hybrid EMT CTCs are positively correlated with metastasis and adverse clinical outcomes, their routine utilization as biomarkers remains restricted by inconsistent capture of marker-shifted cells, lack of standardized phenotypic classification, and insufficient cross-platform analytical validation (Subbalakshmi et al., 2022).

EMT biomarkers potentially lacks standardized detection methodologies which hugely affects their clinical translation. Usage of different antibody panels, cut-off values, and scoring systems for EMT marker assessment often generates inconsistent and non-reproducible results (Batis et al., 2021). To note, the dynamic and reversible nature of EMT tricks the results from the single diagnosis and may promote tumor progression depends in the cellular context (Busch et al., 2016). Hence, there is more research needed in development of standardized detection methodologies to successful clinical translation of EMT biomarkers.

### 5.2 Potential of multi-omics and AI for EMT profiling

Emerging technologies integrating multi-omics (genomic, transcriptomic, proteomic, metabolomic, and epigenomic) data with artificial intelligence (AI) holds transformative potential to overcoming current EMT profiling limitations. It helps in the understanding of comprehensive molecular characterization of multifaceted EMT nature and behaviour rather than the conventional single marker identification which leads to improper results in clinical settings (Khalili-Tanha and Shoari, 2025). This synergistic mechanism is mechanistically necessary as EMT cannot be measured satisfactorily at any single molecular surface: transitions between states are transcriptionally modulated, plasticity stabilized by epigenetic mechanisms, plasticity signaled by proteomic changes and adaptive response to invasion and invasion resistance by metabolomic readings.

Integration of multi-omics approaches along with spatial transcriptomics helps us in the identification and dissection of EMT heterogeneity within the intact tissues (Tran et al., 2025). It hugely offers a simultaneous measurement of multiple molecular layers of EMT and enables us to observe how the local tumor-immune and stromal interactions provide mechanistic insights and drive or restrain the EMT phenotypic plasticity. Precise characterization of EMT signatures in tumors could be benefitted with the help of integrative spatial transcriptomics with deep learning approaches via analysing the single-cell resolution mapping of gene expression patterns in the TME (Liu X. et al., 2024).

Advanced machine learning algorithms helps analyze complex multi-omics datasets which are unable to studied using the conventional methods. Notably, the graph neural networks (GNN), integrates the complex heterogeneous multi-omics data and precisely predicts the cancer subtypes at molecular level (Alharbi et al., 2025). While the deep learning approaches such as the convolutional neural networks (CNN) and transformer models precisely identifies the EMT-associated features in histopathological images and radiological scans (Zhang J. et al., 2025). As an example, weakly supervised computational pathology systems based on transformers have been shown to be clinically grade diagnostics with the ability to discover molecular markers, indicating the possibility of AI-assisted histopathology to go beyond morphology to the EMT-state inference (Jiang et al., 2024). Moreover, the concept of network-based multi-biomarker discovery methods like FUNMarker and NetAUC stresses the significance of harnessing heterogenous molecular inputs and maximizing predictive efficiency, which is especially important with respect to EMT since it has composite epithelial, mesenchymal, and immune-associated signatures (Li X. et al., 2021; Li X. Y. et al., 2022).

While looking at the therapeutic landscape, the integrative AI models helps in the identification of accurate prediction of drug sensitivity towards both targeted and combinatorial therapies via understanding of EMT-related events (Niazi and Mariam, 2025). Presently, the explainable AI is attracting more interest due to their significant prediction of therapeutics activity and in decision making in clinical settings, which could be harnessed for analysing the EMT phenotypic evolution in tumors (Mao et al., 2025). Despite these advances in multi-omics and AI are available for EMT mapping, there are still challenges such as the extensive training cohorts, data harmonization, reproducibility, privacy, algorithmic bias and computational facilities limits the translational efficacy and clinical implementation and more research should be focussed addressing these limitations.

### 5.3 Integrating EMT inhibition into precision oncology

Firstly, more novel stratification paradigms are heavily required for the EMT-targeted precision oncology. Unlike, the conventional mutation-guided stratification models which specifically analyse genetic alterations and suggest targeted drugs, the EMT is context-sensitive phenotype that alters their phenotypic plasticity based on the TME (Subhadarshini et al., 2023). Thus, a dynamic stratification model is needed to address these and to promote EMT-targeted therapies.

Multiplexed companion diagnostics strategies such as the immuno-histochemistry, gene expression profiles and circulating biomarker panels helps in the clinical translation of EMT-targeted therapies by stratifying patients into distinct categories like epithelial-predominant, hybrid, and mesenchymal-predominant based on the EMT status (Khalili-Tanha and Shoari, 2025). The most recent instances of pan-cancer research revealing new prognostic and immunotherapeutic biomarkers, like adhesion G protein-coupled receptor G6 (ADGRG6), also justify the incorporation of molecular and immune-context characteristics into stratification models that can be expanded to include used EMT-state-dependent therapeutic decision-making (Zhu et al., 2025). Likewise, multi-lncRNA prognostic signatures linked to immunotherapy response, including cuproptosis-associated lncRNA models, shows the emerging potential of integrative transcriptomic biomarkers that may complement EMT-based classification systems (Gong et al., 2024). Longitudinal real-time monitoring of EMT status hugely helps in the identification of enable adaptive therapeutic strategies in clinical settings (Allam et al., 2020).

Precise profiling of EMT status is an important strategy for optimizing patient for EMT targeted treatments in clinical settings. Notably, EMT status may effect treatment response not only by promoting chemoresistance, but also by altering ferroptosis sensitivity, since EMT-high/ZEB1-high states can boost PUFA-phospholipid burden and lipid peroxidation, while concurrent stimulation of GPX4-or FSP1-dependent antioxidant defenses determines whether this vulnerability can be pharmacologically exploited (Zhang et al., 2024). Towards immunotherapy treatments, the EMT status helps us in the identification of immune checkpoint blockade outcomes in patients, due to their role in immunosuppressive TME (Shi et al., 2023).

Development of pharmacologically optimized inhibitors selective towards EMT transcription factors regulators such as SNAIL, TWIST, ZEB has limited translational potential and needs more attention. Also, TGF-β inhibitors showed pleiotropic effects and dose-limiting toxicity towards the EMT-targeted therapies (Huynh et al., 2019). EMT phenotypes are reported to sensitize to MAPK inhibitors in resistant melanomas indicating their combination treatment potential (Tang et al., 2020a). Furthermore, integrating EMT inhibitors with next-generation targeted therapies such as immunotherapies, ferroptosis inducers and non-coding RNAs indicates a promising hope for the precision oncology of EMT-targeted therapies.

Translationalally, the future of EMT-targeted oncology will not rely on determining how EMT is a universal therapeutic endpoint but on establishment of when, where and in whom of the clinical actionability of EMT-directed intervention. This involves the use of biomarkers in the selection of patients, longitudinal analysis of state transitions in EMT, rational combinations with immunotherapy, ferroptosis-based classes, targeted therapeutics, or RNA-directed therapies. Consequently, future effective clinical translation will require the implementation of EMT status in adaptive treatment models as opposed to EMT being a fixed binary phenotype.

## 6 Conclusion

EMT is one of the major cancer cell survival strategies that enable tumor cells to acquire invasive, metastatic, and/or therapy-resistant characteristics. Instead of being a binary process, EMT is a continuous dynamic process, where intermediate or transitional states can indirectly select the most aggressive and treatment refractory tumor states. This plasticity is important to understand to elucidate tumor heterogeneity, immune evasion, and disease relapse. The development of molecular profiling has further revealed the complex feature of EMT regulation networks comprising of transcription factors, signaling cascades and epigenetic regulation. The inclusion of these lessons in the biomarker discovery has created new frontiers in identifying the EMT states using circulating tumor cells, exosomes, and gene expression patterns. These biomarkers have potential in predicting metastasis, tracking therapeutic efficiency and personalized intercession.

As a treatment perspective, EMT targeting is both difficult to achieve and progressively available. Anti-EMT-inducing pathway strategies, re-epithelialization strategies to reprogram mesenchymal cells, and EMT-inducing cell therapy sensitisation strategies are becoming increasingly popular. EMT pathways targeted with nanoparticle-based formulations and exosome-derived carriers with novel drug delivery systems can be more precise in therapeutic delivery and avoid systemic toxicity by structuring, coupled with prolonged modulation.
